# Local adaptation to hosts and parasitoids shape *Hamiltonella defensa* genotypes across aphid species

**DOI:** 10.1098/rspb.2022.1269

**Published:** 2022-10-26

**Authors:** Taoping Wu, David Monnin, Rene A. R. Lee, Lee M. Henry

**Affiliations:** School of Biological and Behavioural Sciences, Queen Mary University of London, London E1 4NS, UK

**Keywords:** defensive symbiosis, *Hamiltonella defensa*, parasitoid, local adaptation

## Abstract

Facultative symbionts are common in insects and can provide their hosts with significant adaptations. Yet we still have a limited understanding of what shapes their distributions, such as why particular symbiont strains are common in some host species yet absent in others. To address this question, we genotyped the defensive symbiont *Hamiltonella defensa* in 26 aphid species that commonly carry this microbe. We found that *Hamiltonella* strains were strongly associated with specific aphid species and that strains found in one host species rarely occurred in others. To explain these associations, we reciprocally transferred the *Hamiltonella* strains of three aphid species, *Acyrthosiphon pisum*, *Macrosiphoniella artemisiae* and *Macrosiphum euphorbiae*, and assessed the impact of *Hamiltonella* strain on: the stability of the symbiosis, aphid fecundity and parasitoid resistance. We demonstrate that the *Hamiltonella* strains found in nature are locally adapted to specific aphid hosts, and their ecology: aphids tend to carry *Hamiltonella* strains that are efficiently transmitted to their offspring, non-lethal, and that provide strong protection against their dominant parasitoid species. Our results suggest that facultative symbiont distributions are shaped by selection from natural enemies, and the host itself, resulting in locally adapted symbioses that provide significant benefits against prevailing natural enemies.

## 1. Introduction

Many insects harbour intracellular bacteria that profoundly influence their biology. This includes ancient obligate associations where the symbionts provide insects with essential nutrients and are strictly vertically transmitted through the host matriline. More widespread are heritable facultative symbionts, which are not essential for host survival but can provide important benefits such as expanding their hosts' diet breadth, or conferring resistance to natural enemies, heat stress and even pesticides [1–3]. Facultative symbionts can horizontally transfer between host lineages [4,5] and are often strongly non-randomly associated with particular host species or populations [6–9]. This raises the question of what factors determine the distribution of facultative symbionts across host species. One hypothesis is that insects tend to harbour facultative symbionts that are adapted to the ecological niche in which they reside, thus providing the insect with niche specific benefits.

A common benefit provided by facultative symbionts is protection against natural enemies, such as nematodes, pathogenic fungi, viruses and parasitoids [10–14]. It has been hypothesized that pressures from natural enemies may shape the distribution of protective symbionts across host species. If this were true, facultative symbionts may function in an analogous manner to a horizontal gene pool from which insects can sample to rapidly adapt to changing pressures from natural enemies. However, physiological barriers to the horizontal transmission of facultative symbionts have also been identified [15,16], which may limit their spread.

One of the most extensively studied models for defensive symbiosis is the association between aphids and the facultative symbiont *Hamiltonella defensa. Hamiltonella* is known for being able to protect aphids from parasitoid wasp attack using toxins that are encoded on a phage that is integrated into the symbiont's genome [4,17,18]. However, in certain aphid species, *Hamiltonella* can also be costly by decreasing host lifespan and increasing mortality [19,20]. Furthermore, it was shown that *Hamiltonella* genotypes differentially protect against different parasitoid species attacking aphids [15,21]. This opens the possibility that *Hamiltonella* distributions across aphid species may at least in part be shaped by parasitoids: aphids might host the *Hamiltonella* genotypes that are most efficient at protecting them against their most common natural enemies. However, the extent to which different selective pressures contribute to the genetic structure of *Hamiltonella* across host species is currently unknown. To address this question, we genotyped the *Hamiltonella* strains in 412 aphids from 26 species using six housekeeping genes.

Our survey revealed that *Hamiltonella* genotypes are not randomly distributed across aphid species, but rather form strong associations with particular host species. These patterns can be generated by differential retention of aphid–*Hamiltonella* associations that are formed by horizontal transfer. If the aphid–*Hamiltonella* association is initially suboptimal they can evolve towards optimality through selection on hosts to retain, tolerate and maternally transmit symbionts that increase their fitness. Alternatively, selection can act on the symbionts themselves, favouring microbes that spread within their hosts, are more efficiently transmitted, and provide benefits that increase both host and symbiont frequency in subsequent generations. Irrespective of the relative strength of these different selective processes, we predict that *Hamiltonella* strains are locally adapted to specific aphid species and that this adaptation manifests itself as the symbiont's ability to be efficiently maternally transmitted and to contribute positively to its host's fitness. To test this hypothesis, we experimentally manipulated the infections status of three aphid species—*Acyrthosiphon pisum*, *Macrosiphoniella artemisiae* and *Macrosiphum euphorbiae*—that harbour specific *Hamiltonella* strains using reciprocal transfection. We then measured traits that we expect to be crucial to the evolutionary success of aphid–*Hamiltonella* associations: the efficiency with which the symbiont is maternally transmitted and its impact on host fecundity and resistance to parasitoid attacks.

## 2. Results

### (a) *Hamiltonella*–aphid associations are not random

**Figure 1.**
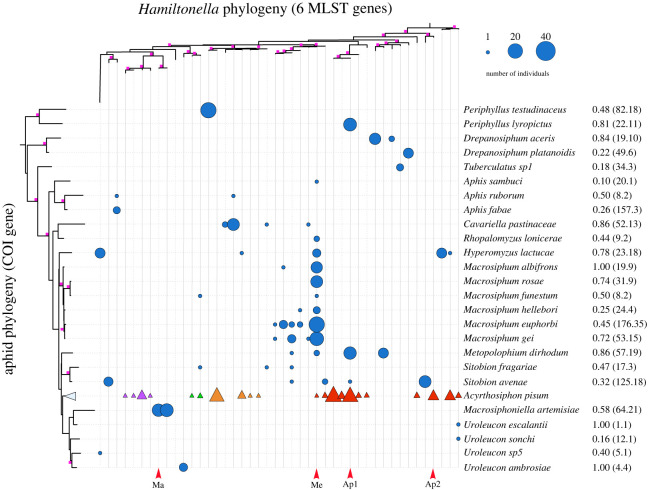
Interaction matrix of *Hamiltonella* genotypes (top phylogeny) occurring in aphid species (left phylogeny). Squares on the phylogeny nodes denote branch support greater than 50. Bubble size corresponds to the number of times an aphid species was found harbouring a particular *Hamiltonella* genotypes. Numbers to the right of the species names indicate the proportion of aphids carrying *Hamiltonella*, and the total number of aphids screened and those included in the matrix in brackets. Triangles represent the major *Hamiltonella* lineages associated with the pea aphid (*A. pisum*) plant-adapted biotypes; *Ononis* spp. (purple), *Trifolium* spp. (green), *Lotus pedunculatus* (orange) and *Medicago sativa* (red) identified in [7]. The *A. pisum* data are not included in analyses. Arrows at the bottom of the matrix denote *Hamiltonella* lineages used in the reciprocal transfection experiments. (Online version in colour.)

We found that *Hamiltonella* genotypes are strongly non-randomly distributed among aphid species (figure 1). This is similar to what has been observed in the pea aphid, *A. pisum*, which is a complex of genetically differentiated plant-adapted ‘biotypes' that also host-specific *Hamiltonella* genotypes [7]. In figure 1, we have included the major *Hamiltonella* lineages found in the *A. pisum* complex (identified in [7]) to show how they are related to *Hamiltonella* genotypes found in other aphid species, as well as to show the phylogenetic placement of our experimental lines (i.e. Ap1 and Ap2). The *A. pisum* data are not included in our analyses. We analysed the importance of *Hamiltonella* genotype, aphid species, aphid and *Hamiltonella* phylogeny, and the interactions between these variables, in explaining the symbionts distribution across aphid species (electronic supplementary material, table S1). The only factor explaining the presence of *Hamiltonella* was the host and symbiont co-phylogeny (Bayesian general linear model (BPMM): posterior mode = 0.56, credible interval = 0.16–0.92; electronic supplementary material, table S1). This means that related aphid species tend to harbour a small number of related *Hamiltonella* genotypes. An example of this can be seen in the genus *Macrosiphum* (figure 1), where all species sampled were predominantly associated with a single clade of *Hamiltonella*, with one dominant genotype.

### (b) Mechanisms explaining *Hamiltonella*–aphid associations

**Figure 2.**
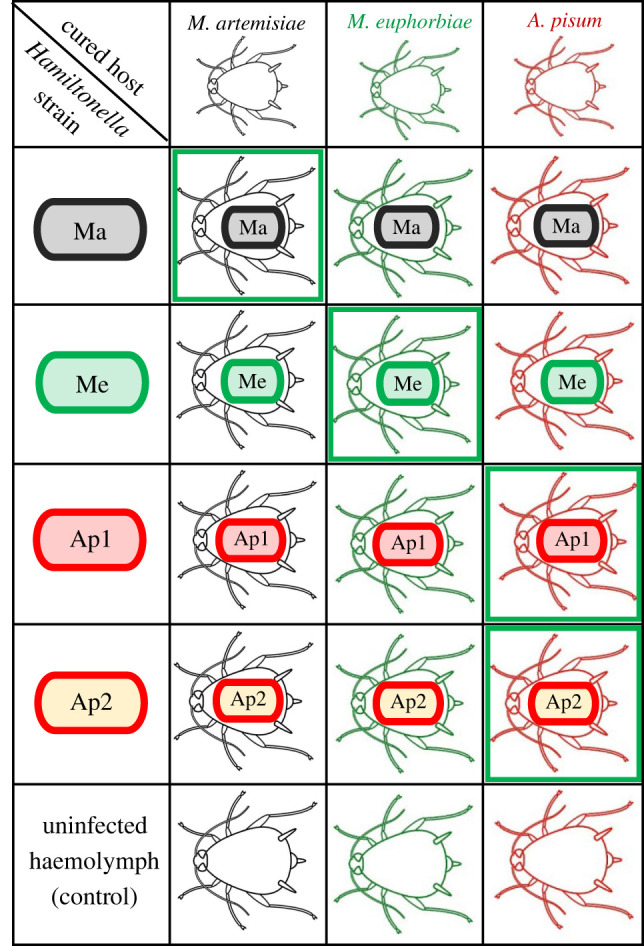
Cross-infection experimental design. We cured two clones each from three aphid species (*Macrosiphoniella artemisiae*, *Macrosiphum euphorbiae* and the *Medicago* biotype ­of *A. pisum*). Each clone was then reinfected with the symbiont genotype(s) it normally hosts in nature, i.e. its native genotype(s) (bold green boxes), and one of three non-native genotypes, or left cured as a control. (Online version in colour.)

To study the factors shaping host–symbiont associations, we focused on three aphid species (two clones each) that maintain strong relationships with specific *Hamiltonella* genotypes: *Macrosiphoniella artemisiae*, *Macrosiphum euphorbiae* and the *Medicago* biotype of *A. pisum*. We studied four *Hamiltonella* strains: Ma is found exclusively in *M. artemisiae*; Me is the dominant strain associated with *M. euphorbiae* and all other *Macrosiphum* species surveyed; and Ap1 and Ap2 from *A. pisum*, one from each of the two major clades of *Hamiltonella* associated with the *Medicago* biotype (identified in [7]) (figure 1). To determine why each aphid species tends to harbour the *Hamiltonella* genotype(s) it does, rather than one of the genotypes found in the other species, we experimentally established aphid clones of each species carrying, its native *Hamiltonella* genotype(s), and those from other aphid species (i.e. non-native genotypes). We then assessed the impact of each *Hamiltonella* genotype–aphid species combination on (i) the frequency the symbiont is passed to offspring (i.e. stability of maternal transmission), (ii) the fecundity of aphids, and (iii) protection against the parasitoid most commonly attacking the aphid species in nature. The reciprocal crosses resulted in 30 aphid clone–*Hamiltonella* genotype treatments: three aphid species * two clones * five infection status (four *Hamiltonella* strains + one *Hamiltonella*-negative line) (figure 2). We found very little difference between clones of the same species so results from both clones are presented and discussed together (results on individual clones can be found in the electronic supplementary material). All lines were confirmed to carry *Hamiltonella* both before and after experiments by testing sibling aphids using diagnostic PCR (see methods).

### (c) *Hamiltonella*–aphid associations can be unstable and lethal to hosts

First, we assessed the stability of each experimentally established *Hamiltonella*–aphid combination by determining how often the symbiont is maternally transmitted to offspring. To do this, we tested for the presence of *Hamiltonella* in at least three offspring per newly infected female line for at least five generations using diagnostic PCR.

Overall, we find that native associations are more viable, in that they are less prone to symbiont loss or aphid lethality, than non-native ones ($\chi_{1}^{2}=43.6$, *p* < 0.001).

**Figure 3.**
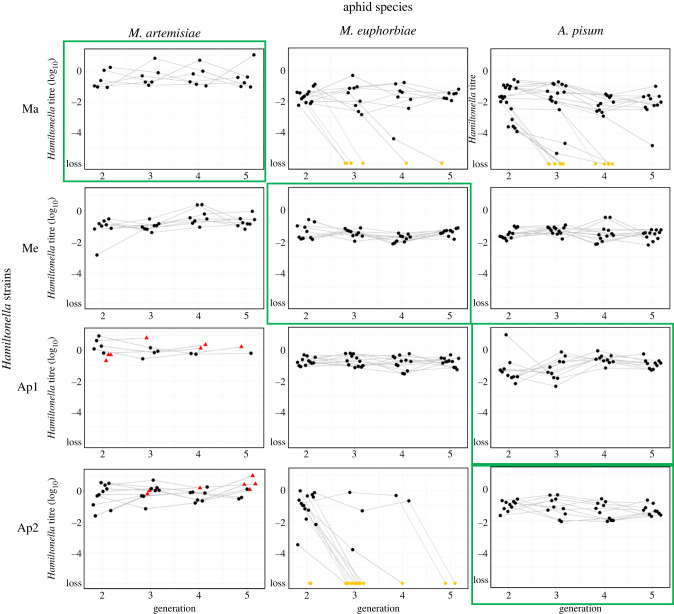
Changes in *Hamiltonella* titres across host generations following injection. Dots represent a single offspring (1st of 3) screened from independent aphid lines injected with *Hamiltonella* with grey lines connecting the same aphid lineage across generations. Red triangles represent lineages that went extinct. Yellow dots at the bottom of the panels indicate the screened aphid no longer contained the symbionts (i.e. the symbiosis was lost). Bold green squares around panels highlight native host–symbiont combinations. Aphid lines that died in the same generation they were injected are not shown here. For the full data on symbiont losses in all offspring tested, line extinctions and initial establishment of infections, see electronic supplementary material, table S2. (Online version in colour.)

Out of the 12 (24 including both clones) *Hamiltonella*–aphid species associations, three (six including both clones) were found to be unstable, in that the symbiont was not perfectly transmitted to offspring (figure 3; electronic supplementary material, table S2). In these cases, *Hamiltonella* was often rapidly lost from the clonal line: by generation 5, 100% (16/16) of *M. euphorbiae* lines injected with the *Hamiltonella* strain Ap2 had been lost (when analysing symbiont strain by host species interactions, Ap2 was found to be significantly less efficiently transmitted than the native Me strain: *z* = 3.2, *p* = 0.022). Similarly, *Hamiltonella* strain Ma was lost in both *A. pisum* (10/21 = 48%) and *M. euphorbiae* (6/13 = 46%) by generation 5 (though in these cases, the differences with the native strains were not statistically significant: *z* = 2.0, *p* = 0.446 and *z* = −1.9, *p* = 0.505, respectively). In all other combinations, no *Hamiltonella* losses were observed (see electronic supplementary material, table S3 for full results).

In two additional associations, *Hamiltonella* strains Ap1 and Ap2 injected into *M. artemisiae*, symbionts were not lost, but rather led to early host death and a high rate of line extinction (red triangles in figure 3; electronic supplementary material, table S2): within two generations after the injection, 76% (16/21) of Ma clones infected with Ap1 (compared to the native Ma strain: *z* = −2.7, *p* = 0.091) and 50% (8/16) of those infected with Ap2 (compared to the native Ma strain: *z* = −2.4, *p* = 0.195), died. All Ap1-*M. artemisiae* and Ap2-*M. artemisiae* lines were extinct by generations 6 and 8, respectively. All unstable aphid–*Hamiltonella* associations, and those that cause increased host mortality, were not normally found in nature (i.e. non-native associations) (figure 3; electronic supplementary material, table S2).

### (d) Infection status impacts fecundity

**Figure 4.**
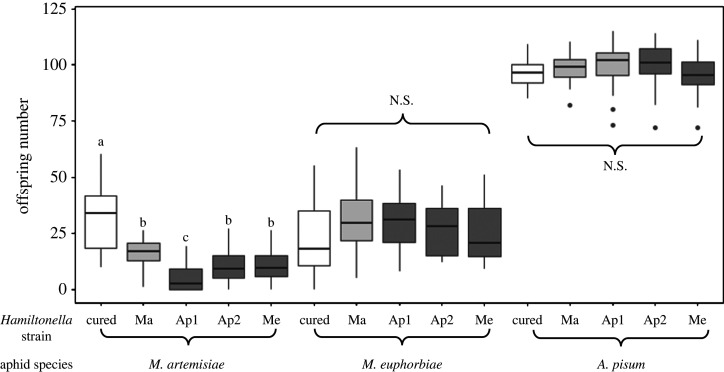
Impact of infection status on fecundity of *M. artemisiae, M. euphorbiae* and *A. pisum*. *Hamiltonella*-negative lines are shown in white, native associations in light grey and non-native associations in dark grey. Fecundity assays were only conducted on a single aphid clone per species.

We then assessed the impact of infection status on aphid fecundity (figure 4). All fecundity assays were only conducted on a single clone per aphid species (Mug3 in *M. artemisiae*, PotG in *M. euphorbiae*, ApY in *A. pisum*). The nature of the association had an effect on fecundity ($\chi_{2}^{2}=8.2$, *p* = 0.016): it was significantly higher in native associations than in non-native associations (*z* = 2.4, *p* = 0.044). There was a tendency for cured aphids to have a higher fecundity than aphids infected with non-native strains (*z* = −2.2, *p* = 0.067), and no difference between cured aphids and aphids infected with native strains (*z* = −0.9, *p* = 0.630).

Analysing symbiont strain by host species interactions revealed that *Hamiltonella* infections status had a significant impact on the fecundity of *M. artemisiae* but not the other two aphid species (electronic supplementary material, table S3): cured *M. artemisiae* individuals had a higher fecundity than those infected with Ma (*z* = 4.2, *p* < 0.001), Ap1 (*z* = −11.7, *p* < 0.001), Ap2 (*z* = −7.4, *p* < 0.001) and Me (*z* = 7.6, *p* < 0.001). Ap1 had a particularly detrimental effect, as it significantly reduced fecundity compared to the native Ma strain (*z* = −7.1, *p* < 0.001) (figure 4). Both Ap1 and Ap2 eventually led to line extinction when carried by *M. artemisiae* (red triangles, bottom left panels of figure 3).

### (e) Infection status impacts resistance against parasitoids

We tested whether aphids are associated with *Hamiltonella* strains in nature that provide high degrees of protection against the parasitoid species that most commonly attack them. To test this hypothesis, we first surveyed the parasitoid species attacking the three aphid species used in this study. Second, we assessed the capacity of each *Hamiltonella* strain to protect aphids against the parasitoid species most commonly attacking them in the UK.

A total of 238 aphid–parasitoid associations were identified (table 1; electronic supplementary material, tables S4–S6) using a combination of deep-coverage barcoding of aphid mummies and Sanger sequencing of parasitoids that emerged from morphologically identified aphids. All three aphid species in our study were predominantly attacked by a single parasitoid species: *Aphidius absinthii* in *M. artemisiae* (100%), *Aphidius ervi* in *A. pisum* (89%) and *Aphidius rhopalosiphi* in *M. euphorbiae* (67%). One parasitoid species, *A. ervi*, was found to attack both *A. pisum* and *M. euphorbiae* at lower frequencies (11%).

**Table 1.** Aphid–parasitoid association list. Illumina sequencing was used for simultaneous identification of the aphid and parasitoid. Sanger sequencing was used to identify parasitoids emerging from aphids identified using morphology and host plant. Asterisks in the proportion column indicate the dominant parasitoid of each aphid. More information on collection can be found in electronic supplementary material, tables S4–S6.

aphid species	parasitoid species	Illumina	Sanger	total	proportion
			2019–2022		
*Macrosiphoniella artemisiae* (*n* = 80) sampling sites = 6	*Aphidius absinthii*	6	74	80	100.0%*
*Macrosiphum euphorbiae* (*n* = 66) sampling sites = 8	*Aphidius ervi*	7		7	10.61%
	*Aphidius matricariae*	2		2	3.03%
	*Aphidius rhopalosiphi*	30	5	35	53.03%
	*Ephedrus californicus*		4	4	6.06%
	*Ephedrus lacertosus*	1		1	1.52%
	*Praon volucre*		3	3	4.55%
	*Praon* spp.	4	5	9	13.64%
	*Aphidius rosae*	2		2	3.03%
	*Areopraon silvestre*	1		1	1.52%
	*Monoctonus leclanti*	1		1	1.52%
	*Toxares deltiger*	1		1	1.52%
*Acyrthosiphon pisum* (Medicago) (*n* = 92) sampling sites = 5	*Aphidius banksae*		12	12	13.04%
	*Aphidius ervi*		72	72	78.26%*
	*Aphidius eadyi*		1	1	1.09%
	*Aphidius rhopalosiphi*		1	1	1.09%
	*Aphidius microlophii*		3	3	3.26%
	*Praon barbatum*		3	3	3.26%

We experimentally tested the impact of *Hamiltonella* status on resistance against parasitoids, by exposing each aphid species to the dominant parasitoid species attacking it. In general, we found that the nature of the association had a significant effect on parasitoid resistance ($\chi_{2}^{2}=118.7$, *p* < 0.001): native host–symbiont pairing provided greater protection from parasitoids than non-native associations (*z* = 9.8 *p* < 0.001). There was no difference, however, between cured aphids and their native associations (*z* = 1.4, *p* = 0.324) or cured and non-native associations (*z* = 0.5, *p* = 0.873).

**Figure 5.**
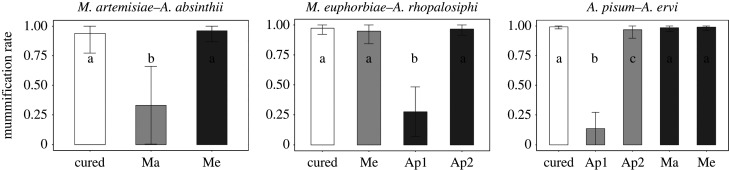
Effect of *Hamiltonella* genotype on protection from parasitoid wasp attack in three aphid species. Each aphid species was exposed to its dominant parasitoid. Protection was determined by the wasp mummification rate: 0 indicates that all aphids resisted the attack, and 1 that all aphids were parasitized (mean ± s.e.). *Hamiltonella*-negative lines are shown in white, native associations in light grey, and non-native associations in dark grey. Only stable experimental lines were assessed. For results on individual clones, see electronic supplementary material, figure S1.

Analysing aphid–parasitoid pairs individually revealed that *Hamiltonella* infection status had a significant effect on mummification rate in all three cases: *M. artemisiae*-*A. absinthii* ($\chi_{2}^{2}=175.6$, *p* < 0.001), *M. euphorbiae*-*A. rhopalosiphi* ($\chi_{3}^{2}=641.5$, *p* < 0.001) and *A. pisum*-*A. ervi* ($\chi_{4}^{2}=1198.7$, *p* < 0.001). All cured aphids were highly vulnerable to attack by their dominant parasitoid, as shown by mummification rates close to 1 (figure 5; electronic supplementary material, figure S1 for individual clones). When comparing the protection conferred by different symbiont strains, we found that in three of four cases, the native *Hamiltonella* genotype(s) provided the greatest degree of protection against the aphids' dominant parasitoid: compared to other symbiont strains, Ma provided *M. artemisiae* with the highest degree of protection against *A. absinthii* (*z* = 8.7, *p* < 0.001), and both Ap1 and Ap2 protected *A. pisum* against its common parasitoid *A. ervi* (Ap1: *z* = −11.8, *p* < 0.001, Ap2: *z* = −2.9, *p* = 0.032), although one strain (Ap1) provided significantly greater protection (*z* = −13.7, *p* < 0.001). By contrast, Me, the *Hamiltonella* strain most commonly found in *M. euphorbiae*, did not provide its host with any protection against *A. rhopalosiphi*. In *M. euphorbiae*, only the *A. pisum* derived Ap1 (*z* = −13.1, *p* < 0.001) provided protection against the parasitoid *A. rhopalosiphi*. In fact, the Me *Hamiltonella* genotype did not provide protection against any parasitoid in any of the host backgrounds (see electronic supplementary material, table S3 for full results).

## 3. Discussion

### (a) Explaining the non-random genetic structure of *Hamiltonella*

Our survey revealed that aphid species tend to form strong relationships with a single, or a few closely related, *Hamiltonella* strain(s). This is similar to what has been observed in pea aphid biotypes, where the plant-adapted aphids feeding on *Lotus*, *Medicago* and *Ononis*/*Melilotus* carry specific strains of *Hamiltonella* [7]. We also find intermittent cases where the same *Hamiltonella* genotype is carried by unrelated host species, which support previous finding that the symbiont is occasionally horizontally transferred between species [4]. This suggests that a combination of horizontal transfer and selection explains the observed patterns of *Hamiltonella* infections across aphid species. It has been proposed that the facultative symbiont distributions of aphids may be the by-product of selection in response to attack by natural enemies [14], seasonal changes [22] and cost-benefit trade-offs [15,23,24]. However, the mechanisms explaining the strong genetic structure of *Hamiltonella* strains across aphid species have not been explored until now.

**Figure 6.**
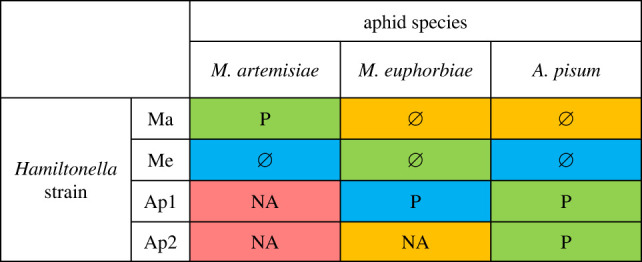
Summary of aphid–*Hamiltonella* experimental crosses. Native associations are shown in green background. Non-native associations are shown in blue unless they are unstable (yellow) or lethal (red). An association is considered unstable or lethal as soon as loss of the symbiont or extinction of the aphid line, respectively, occurred during our experiment. ‘P' indicates protection against the most common parasitoid, ‘Ø' indicates the absence of protection, and ‘NA' indicates that the level of protection was not assessed, due to the experimental association being unstable or lethal. (Online version in colour.)

Overall, we found that native aphid–*Hamiltonella* associations were more stable, less costly to host fecundity, and provided greater protection against parastioids, compared to non-native ones. In nearly all combinations not normally found in nature, the symbiont strain was poorly adapted to either the aphid species or to the most common parasitoid species attacking the aphid. More specifically, we identified three selective filters that play a role in shaping the natural patterns of *Hamiltonella* infections across host species: (i) symbiosis instability, (ii) host lethality and (iii) lack of protection against the most common parasitoid of the host (summarized in figure 6). In the case of the *A. pisum*-Ma association, both instability and lack of protection apply. By contrast, in native associations, the symbionts were always both efficiently vertically transmitted, avirulent, and in most cases, offered significant protection against the insects' most common parasitoid enemy. This demonstrates that *Hamiltonella* genotypes tend to be locally adapted to an aphids' internal and external environment, i.e. to the aphid (host–symbiont genotype–genotype interaction) and to the aphid's ecology (selection from parasitoids).

### (b) Adaptation to internal factors

We assessed the compatibility of host–symbiont combinations by testing how reliably they transmit *Hamiltonella* to their offspring, and the fitness effects of different symbiont strains. Instability and lethality phenotypes were relatively consistent within clones of the same species, which suggests *Hamiltonella* genotypes were incompatible with certain host species (electronic supplementary material, table S2).

Studies on pea aphids have shown that facultative symbionts are more frequently lost during maternal transmission when transferred between host biotypes [25,26]. The probability of losses has also been shown to be influenced by the relatedness of host species [27–29]. We found that all native host–symbiont combinations were perfectly maternally transmitted under laboratory conditions. Similar results have been cited for pea aphids; however, in other aphid species, such as in *Aphis craccivora*, maternal transmission of *Hamiltonella* is high but imperfect [30–32]. Imperfect transmission of facultative symbionts may also be exacerbated during sexual reproduction [33], and impacted by abiotic changes, such as temperature fluctuations [34].

Carrying facultative symbionts can also be costly in some host species, as a result of consuming a portion of the host's resources [20,24,35], or possibly through competing with *Buchnera,* the primary symbiont. However, cases of very strong virulence such as the ones we observed (Ap1 and Ap2 in *M. artemisiae*) have rarely been reported. Our findings are consistent with a previous study showing that *Hamiltonella* can be highly deleterious in *Aphis fabae*, with a decrease in fecundity up to 80% in certain host–symbiont genotype combinations [36]. As with previous studies, we find that *A. pisum* can harbour diverse *Hamiltonella* strains with few costs to fecundity [37,38], and *M. euphorbiae* followed a similar trend [39], possibly even exhibiting a marginal, yet not significant, increase in fecundity when carrying *Hamiltonella*. Although previously thought to be largely benign, our results support the growing evidence that *Hamiltonella* infections can exhibit a wide range of effects on hosts—from benign to strongly virulent—with the outcome being determined by specific host by symbiont strain interactions. Taken together, these results suggest the host–symbiont associations found in aphids are the product of coevolution for decreased symbiont virulence and increased stability of maternal transmission. Furthermore, our results indicate that host–symbiont genetic incompatibilities are a major factor dictating the retention of symbiont genotypes across aphid species.

### (c) Adaptation to external factors

Parasitoids are common natural enemies of aphids, and therefore a strong selective pressure for the evolution of resistance. Previous studies have shown that *Hamiltonella* strains provide specific protection against two distantly related species of parasitoids, *Aphidius ervi* and *Aphelinus abdominalis* [21]. Given that parasitoids tend to be highly host-specific [40], we hypothesized that they may be a major force shaping protective symbiont genotype distributions across aphid species. We found that aphids tend to harbour *Hamiltonella* strains that provide them with at least some, to strong, levels of protection against the most common parasitoid species attacking them [1,41]. This supports our hypothesis that selection from parasitoids has resulted in aphids tending to carry symbiont strains that provide them with protection. Furthermore, we found that *Hamiltonella* strains can provide highly specific protection even against closely related parasitoid species. For example, the Ma strain of *Hamiltonella* provided protection against *A. absinthii*, but not *A. ervi*, which are both *Aphidius* parasitoids.

In some cases, different aphid species share the same *Hamiltonella* genotype. This is most prominent in the *Macrosiphum* genus but can also occurred in more distantly related species, such as *A. pisum* and *Periphyllus lyropictus*. It is currently unclear why aphid species share the same *Hamiltonella* stains in some cases, but not in others. It would be interesting to know whether aphids that share *Hamiltonella* strains also share the same parasitoids, and potentially use the symbiont strain as a common resource to protect against the same natural enemy.

Our results suggest that parasitoids are an important selective pressure shaping the distribution of *Hamiltonella* genotypes found in nature. Future studies could test whether pressures from natural enemies are responsible for the genetic structuring of defensive symbionts in other systems. For example, in pea aphids, the *Hamiltonella* strains occurring in different biotypes may be the product of selection from different parasitoid species, or perhaps populations within a species, that are adapted to attacking the different aphid biotypes.

### (d) Puzzling associations

For the most part, our results help explain why some aphid–*Hamiltonella* associations are found in nature and not others. However, the association of *M. euphorbiae* with Me, rather than Ap1, remains puzzling. Both strains are apparently well suited to the aphid, as they are both stable and avirulent, but contrary to Ap1, Me is not able to protect *M. euphorbiae* against *A. rhopalosiphi*, which is a common parasitoid attacking the aphid (a similar result was reported in *Aphis craccivora* [42]). As noted above, vertical transmission rates may differ in nature versus laboratory conditions, and this may help explain why Ap1 is not hosted by *M. euphorbiae*, if Ap1 is not effectively transmitted to offspring in nature. Alternatively, its absence in *M. euphorbiae* could be explained by the lack of opportunities for horizontal transfers to this host, or it may be an inferior intra-host competitor to the Me strain. However, the prevalence of Me in *M. euphorbiae* (and other *Macrosiphum* species) at such high frequencies (figure 1) is in itself surprising, as we did not find evidence for any benefit conferred to its host.

*Macrosiphum euphorbiae* tends to be attacked by a greater diversity of parasitoid species and is associated with more *Hamiltonella* strains than the other aphid species. The lack of protection conferred by Me is therefore arguably less surprising than in a species consistently attacked by only one parasitoid species (such as *M. artemisiae*). First, based on collections mostly performed on *Geum* spp. and *Gallium aparine* (electronic supplementary material, tables S4 and S5), we found that *M. euphorbiae* was most commonly attacked by *A. rhopalosiphi*. However, it is possible that on other plants, *M. euphorbiae* is attacked by different parasitoids that Me protects against. The rate at which aphids are targeted by a given parasitoid has been shown to vary depending on the host plant [43] For example, *M. euphorbiae* feeding on pepper plants in Spain are more commonly attacked by *Aphidius colemani*, *Praon volucre* and *Aphidius matricariae* [44], whereas in North America potato farms, it is primarily attacked by *Aphidius nigripus* [45]. Second, *M. euphorbiae* can harbour several related *Hamiltonella* strains, and we only tested one of them (the most common, according to our survey). It is possible that other strains provide protection against *A. rhopalosiphi*. As a result, we cannot exclude the possibility that the *Hamiltonella* association found in *M. euphorbiae* is, to some extent, influenced by symbiont-parasitoid associations that we did not test in our study.

The *M. euphorbiae*-Me association might also be explained by adaptation to external factors unrelated to parasitoids. It might, for example, protect against non-parasitoid natural enemies, e.g. lady beetles, as reported in [46], or suppress the immune system of host plants [47]. It is therefore possible that although Me did not seem to improve *M. euphorbiae* performance on broad beans, it may increase its fecundity on other host plant species, including its native host plant *Gallium*.

## 4. Conclusion

We have shown that aphid species tend to host specific strains of facultative symbionts. These patterns persist despite ongoing horizontal transmission of *Hamiltonella*, which can be seen through the same symbiont genotypes occurring at low frequencies in different host species. When challenged with parasitoids, we find that the symbiont strain an aphid carries is typically well suited to protect against the parasitoid species most commonly attacking them. Attack frequencies from parasitoids may therefore impose an initial selective pressure that results in aphids being associated with symbiont strains that confer strong protection. However, we also find that the host itself imposes a strong selective pressure on symbionts; 5 of 8 non-native host–symbiont combinations were either unstable or lethal to the host. This demonstrates that despite ongoing horizontal transfer, many novel aphid–*Hamiltonella* will simply be lost due to instability. However, in some cases, non-native host–symbiont combinations formed stable, avirulent, beneficial symbioses (e.g. Ap1 in *M. euphorbiae*), and not all unstable combinations were immediately lost; only a fraction of injected lines actually lost *Hamiltonella* while others persisted for generations. It is possible that in some cases, novel symbiont combinations such as these eventually stabilize over a period of coevolution and are retained in populations resulting in strong associations between aphid species and specific symbiont strains. This is particularly likely in scenarios where the benefit of retaining the symbiont for parasitoid protection outweighs the cost of carrying it. It has been suggested that facultative symbionts may function as a horizontal gene pool that insects can draw from to adapt to changing environments. Although the rate of horizontal transfer of *Hamiltonella* is currently unknown, our findings support the potential for rapid adaptation through symbiont acquisition; despite not occurring in *M. euphorbiae*, the Ap1 strain of *Hamiltonella* efficiently protects against its main parasitoid, *A. rhopalosiphi*. As parasitoid frequencies change over time, these genotypes, if protective, might allow for rapid adaptation and approach fixation. Taken together, our results provide strong support for the local adaptation of aphid species and specific *Hamiltonella* strains and suggest that changes in pressure from parasitoids may be rapidly addressed by the acquisition of new symbiont strains.

## 5. Material and methods

### (a) Patterns of *Hamiltonella*–aphid associations

#### (i) Aphid collection and identification

Aphids were collected in the UK between 2011 and 2019 by beating plants over a white tray or manually removing them from plants, before being placed in 100% ethanol. Resampling of the same aphid clones was minimized by separating collections from the same plant species by at least 10 m. Aphids were identified by DNA barcoding based on data from [48] and confirmed using morphological examination following [49]. Genomic DNA was extracted from individual specimens using DNeasy Blood and Tissue kits (QIAGEN, Venlo, Netherlands) and then we amplified an approximately 700 bp DNA fragment of the cytochrome c oxidase I (COI) mitochondrial gene from the DNA using the Lep F and Lep R primers. We sequenced the amplicons in the forward direction (full details of PCR conditions and primer sequences are provided in electronic supplementary material, table S7). DNA sequences were aligned with MUSCLE (https://www.ebi.ac.uk/Tools/msa/muscle/). Aphids were identified to species by comparing COI sequence data to the online databases BOLD (http://www.boldsystems.org/) and GenBank using BLAST. Information on collected aphids are provided in electronic supplementary material, table S8.

### (b) Hamiltonella screening and genotyping

*Hamiltonella* was detected using diagnostic PCR based on the 16S ribosomal RNA gene (electronic supplementary material, table S7) amplified from the whole body aphid DNA extracts. Genotyping was performed using a multilocus sequence-typing (MLST) scheme containing six bacterial housekeeping genes: accD, gyrB, hrpA, murE, recJ and rpoS [50]. PCR products were Sanger-sequenced in the forward direction, and aligned using MUSCLE (https://www.ebi.ac.uk/Tools/msa/muscle/).

#### (i) Phylogenetic reconstruction

Host (COI) and symbiont (MLST) phylogenies were reconstructed using PhyML 3.0 (http://www.atgc-montpellier.fr/phyml/) with 100 standard bootstrap analysis and otherwise default parameter values. *Adelges cooleyi* and a *Hamiltonella* strain of *Bemisia tabaci* were used as outgroups in the host phylogeny and the symbiont phylogeny, respectively.

#### (ii) Bayesian general linear modelling

We used BPMMs with Markov Chain Monte Carlo (MCMC) estimation run in the mixed model package MCMCglmm [51] in R v. 4.2.0 [52]. The occurrence and non-occurrence of *Hamiltonella*–aphid combinations were fitted as a binomial response variable. *Hamiltonella* genotype, aphid species, genotype-species interaction, *Hamiltonella* phylogeny, aphid phylogeny and *Hamiltonella*–aphid cophylogeny were included as random explanatory variables.

The MCMC was run for ten million iterations with a thinning interval of 225 and a ‘burn in' of 100 000. Convergence of the chains was confirmed by visual inspection of the trace plots. We present the results as the posterior modes (PM) with the 95% credible intervals of the estimate.

### (c) Maintenance of aphid lines and experiments

#### (i) Establishment of aphid lines

Aphids belonging to three different species (*A. pisum*, *M. artemisiae*, *M. euphorbiae*) were collected live in the field. Three clonal lines per species were established. Secondary symbionts infection statuses of all aphid clones were tested by 16 s rRNA PCR (electronic supplementary material, table S7). They were found to be positive for *Hamiltonella*, but negative for *Serratia symbiotica*, *Fukatsuia symbiotica*, *Rickettsia *sp., *Rickettsiella *sp., *Spiroplasma *sp. and *Regiella insecticola*. Aphids cured of *Hamiltonella* using selective antibiotics served as ‘recipients’ for transfection. Clonal lines carrying each *Hamiltonella* strain of interest were retained as symbiont ‘donors' for artificial transfections. Clonal lines of aphids were maintained in the laboratory at 15°C with a 16 h light (Sylvania Gro-Lux F36W/GRO-T8 bulb) 8 h dark cycle on a leaf of *Vicia faba* (*A. pisum* and *M. euphorbiae*) or *Artemisia vulgaris* (*M. artemisiae*) embedded in 2% agar in a Petri dish. Leaves were changed weekly.

#### (ii) Antibiotic curing

We used antibiotic treatments to selectively remove *Hamiltonella* without eliminating the primary symbiont *Buchnera aphidicola*, following a protocol adapted from [53]. The antibiotic solution was obtained by mixing 10 mg ml^−1^ of Ampicillin sodium salt, 5 mg ml^−1^ Cefotaxime sodium salt and 5 mg ml^−1^ Gentamicin in water. A single leaf of the host plant was cut and placed in a 0.5 ml Eppendorf tube filled with the antibiotic solution. We placed 10 1- or 2-day-old aphid nymphs on a leaf and left them to feed for five days on antibiotic solution. Surviving aphids were then transferred to a regular Petri dish as described above. We confirmed the antibiotic had removed *Hamiltonella* by testing aphids in the second generation after treatments, and in the sixth generation prior to the start of experiments, using symbiont-specific 16S rRNA primers. The presence/absence/strain of each *Hamiltonella* treatment was reconfirmed after the experiments had been conducted.

#### (iii) *Hamiltonella* transfections

*Hamiltonella*–aphid associations were established using haemolymph injection (figure 2 and table 2). Approximately 0.25 µl of haemolymph was obtained by removing the leg of a naturally infected adult (donor) aphid and then injected into a first instar uninfected (recipient) aphid using a microcapillary needle [15]. Injected aphids were maintained until they reach adulthood and the presence of *Hamiltonella* was checked in three of their late offspring (greater than 10th in birth order, in most cases) through DNA extraction and PCR (as described above). Successful injection lines were kept for a minimum of seven generations before being used in parasitoid experiments, to ensure the stability of the infection [54]. At least 13 independent injection lines per *Hamiltonella*–aphid combination were established (electronic supplementary material, table S2). Their infection status was confirmed both immediately before and immediately after all experiments in which they were involved using 16S rRNA PCR by testing the siblings of the aphids being assayed.

**Table 2.** Aphid–*Hamiltonella* treatment lines obtained through curing and transfection.

species	donor line	recipient line	block 1	block 2
*A. pisum*	—	PeaYc / Pea1c	PeaYc	Pea1c
	Pea1		Ap1→PeaYc	Ap1→Pea1c
	Pea2		Ap2→PeaYc	Ap2→Pea1c
	Mug1		Ma→PeaYc	Ma→Pea1c
	Pot1		Me→PeaYc	Me→Pea1c
*M. artemisiae*	—	Mug3c / Mug20c	Mug3c	Mug20c
	Pea1		Ap1→Mug3c	Ap1→Mug20c
	Pea2		Ap2→Mug3c	Ap2→Mug20c
	Mug1		Ma→Mug3c	Ma→Mug20c
	Pot1		Me→Mug3c	Me→Mug20c
*M. euphorbiae*	—	Pot8c / PotGc	Pot8c	PotGc
	Pea1		Ap1→Pot8c	Ap1→PotGc
	Pea2		Ap2→Pot8c	Ap2→PotGc
	Mug1		Ma→Pot8c	Ma→PotGc
	Pot1		Me→Pot8c	Me→PotGc

### (d) Stability of *Hamiltonella* infections and fecundity effects

#### (i) Stability of Hamiltonella infections

We assessed the stability of newly established *Hamiltonella* infections at generations 2–5 following injection by measuring the relative density of *Hamiltonella* using quantitative PCR on whole aphid DNA extracts. Three 14-days-old aphids per injection line (six injection lines per *Hamiltonella*–aphid combination) were extracted as described above. We used two single-copy genes: one in the aphid nuclear genome (EF 1-α) and one in *Hamiltonella* (dnaK). The quantification was performed on a CFX Connect Real-Time PCR Detection System (BioRad, Hercules, CA, USA). Full details of PCR conditions and primer sequences are provided in electronic supplementary material, table S7. The mean qPCR efficiencies were calculated using a ten-fold series of dilutions from 3.2 × 10^2^ to 3.2 × 10^7^ copies of purified PCR products. The efficiencies were 96.3% for the aphid gene and 89.1% for the *Hamiltonella* gene. Samples were run in triplicates. As the standard deviations between the triplicates of a given samples were below 0.5 cycles, the mean quantification cycle (Cq) values were used to calculate the starting quantities of the genes of interest. For each sample, the starting quantity for the *Hamiltonella* gene was divided by the starting quantity for the aphid gene to obtain the *Hamiltonella* density.

#### (ii) Aphid fecundity

Lifetime fecundity was recorded for 5–36 (approx. 24 on average) adult aphids per infection status and recipient species (Mug3, PotG and PeaY), from either cured lines or newly injected lines (from generation 2 to generation 7 following microinjection). Dishes were checked weekly, all offspring were counted and then removed. Aphids that died prematurely from fungal infection were excluded from the dataset.

### (e) Parasitoid survey and resistance experiment

#### (i) Collection and identification of mummified aphids

Mummified and live aphids were collected in the Greater London area (UK) on five plant genera or species (*Geum* spp., *Galium aparine*, *Medicago sativa* and *Artemisia* spp.) known to host *M. euphorbiae*, *A. pisum* and *M. artemisae* (see electronic supplementary material, table S5 and S6 for full information on mummy collection)*.* Mummified aphids were immediately preserved in 70% ethanol for DNA extraction. Live aphids were kept in the laboratory on a leaf of their host plant for two weeks. Any mummy forming during this time was either preserved for DNA extraction or used to establish laboratory colonies (see below). DNA extraction of mummified aphids was performed as previously described.

To simultaneously identified the aphid and its parasitoid from field collected aphids and mummies, we amplify DNA using universal barcoding primers that target the cytochrome c oxidase subunit I ‘COI' gene, Ill_B_F/HCO2198 [55] (electronic supplementary material, table S7). Tagged PCR products were submitted to Bart's and the London Genome Centre for addition of indices and pooling of libraries. Sequencing was then carried out on a single MiSeq run (paired-end, 2 × 300 bp reads). Samples were analysed using Dada 2 v. 1.16 [56].

#### (ii) Parasitoid resistance experiment

Colonies of three parasitoid species were maintained in the laboratory on host aphid clones that had been cured of *Hamiltonella*: *Aphidius absinthii* on *M. artemisiae* (Mug3c), *A. ervi* on *A. pisum* (PeaYc) and *A. rhopalosiphi* on *M. euphorbiae* (PotGc). Temperature and lighting were the same as for aphids. Prior to the experiment, newly hatched wasps of both sexes were kept together for at least 24 h to allow for mating. Parasitoid females were then individually exposed to 1 s instar larva from the tested aphid line. In case the parasitoid failed to attack the aphid within 10 min, it was discarded. Otherwise, it was transferred to a Petri dish containing 15 s instar aphids (4-day-old instars for *M. euphorbiae* and *A. pisum*; 6-day-old instars for *M. artemisiae*) and kept there for 24 h. After 12 days, the mummification rate was calculated as the number of mummified individuals divided by the number of individuals that were either alive or mummified (non-mummified aphids that died before 12 days were excluded). At least 12 biological replicates were used for every experimental line tested.

### (f) Statistical analyses

Statistical analyses were performed in R v. 4.2.0 [52] using RStudio v. 1.4.1743 [57]. The lme4 (v. 1.1-29) package [58] was used to fit generalized linear mixed models (GLMMs). The multcomp (v. 1.4-19) package [59] was used to perform *post hoc* tests.

The stability and lethality, fecundity and parasitoid resistance datasets were each analysed to assess the overall effects of harbouring a native *Hamiltonella* strain, harbouring a non-native *Hamiltonella* strain, and (when it applies) not harbouring any *Hamiltonella* strain.

Stability and lethality data were analysed using a GLM with a binomial distribution. For each host clone–*Hamiltonella* strain association, the number of successes was defined as the number of lines that were alive and infected at generation 5, and the number of failures was defined as the number of lines that were extinct or had lost their symbiont by generation 5. Both the nature of the association (i.e. native or non-native) and the host species were initially included in the model as explanatory factors. Following model selection, the interaction between these two factors ($\chi_{2}^{2}<0.001$, *p* = 1) and the host species factor ($\chi_{2}^{2}=4.2$, *p* = 0.121) was removed.

Fecundity data were analysed using a GLMM with a negative binomial distribution. The nature of the association (native, non-native or cured, i.e. no association) was included as a fixed effect, and the symbiont strain, host clone and injection line as random effects. *Post hoc* tests were performed to allow for pairwise comparisons.

Mummification rate data were analysed by fitting a GLMM with a binomial distribution. The nature of the association (native, non-native or cured) was included as a fixed effect, and the symbiont strain and host clone as random effects. *Post hoc* tests were performed to allow for pairwise comparisons.

Further tests were conducted to identify the specific host–symbiont interactions that explain the overall patterns. In each case, *post hoc* tests were performed to allow for pairwise comparisons.

Stability and lethality data were analysed using a GLM with a binomial distribution and symbiont strain and host species included as explanatory factors. The host clone factor was initially included in the model, nested within the host species factor, but was found to be not significant ($\chi_{3}^{2}=4.2$, *p* = 0.245) and therefore removed from the final model.

Fecundity data were analysed using a GLM with a negative binomial distribution and infection status and host species included as explanatory factors. The injection line factor was initially included in the model, nested within the infection status factor, but was found to be not significant (LRT $\chi_{60}^{2}=55.5$, *p* = 0.640) and therefore removed from the final model.

As parasitoid resistance was not assessed in all aphid–*Hamiltonella* combinations (due to instability or lethality), our design is not a full factorial one. To assess the effect of particular associations, we therefore analysed the data using three different models, one per aphid species. Each model was a GLMM with a binomial distribution, infection status as a fixed effect and host clone as a random effect.

Fecundity data were analysed independently for each species. Three GLMs were fitted, one (*A. pisum*) with a Poisson distribution, two (*M. artemisiae* and *M. euphorbiae*) with a negative binomial distribution (as overdispersion prevented the use of a Poisson distribution). Infection status was included in the model as a fixed effect, and injection line was nested within infection status.

Mummification rate data were analysed independently for each species, by fitting three GLMMs with binomial distributions. Infection status was included as a fixed effect, and clone as a random effect. *Post hoc* tests were performed after model selection.

## Data Availability

Data and scripts are available online: https://doi.org/10.5281/zenodo.7097415 [60]. The GenBank accession numbers for Sanger sequences determined in this study are ON928985 to ON929011 (Aphid COI), ON931787 to ON932056 (*Hamiltonella* MLST), ON993437 - ON993619 (Parasitoid COI). The GenBank accession number for the raw data of Illumina sequencing used in this study is PRJNA863469. The data are provided in electronic supplementary material [61].

## References

[1] Oliver KM, Degnan PH, Burke GR, Moran NA. 2010 Facultative symbionts in aphids and the horizontal transfer of ecologically important traits. Annu. Rev. Entomol. **55**, 247-266. (10.1146/annurev-ento-112408-085305) 19728837

[2] Russell JA, Moran NA. 2006 Costs and benefits of symbiont infection in aphids: variation among symbionts and across temperatures. Proc. R. Soc. B **273**, 603-610. (10.1098/rspb.2005.3348) PMC1560055 16537132

[3] Li Q, Sun JX, Qin YG, Fan J, Zhang Y, Tan XL, Hou ML, Chen JL. 2021 Reduced insecticide susceptibility of the wheat aphid *Sitobion miscanthi* after infection by the secondary bacterial symbiont *Hamiltonella defensa*. Pest Manag. Sci. **77**, 1936-1944. (10.1002/ps.6221) 33300163

[4] Russell JA, Latorre A, Sabater-Muñoz B, Moya A, Moran NA. 2003 Side-stepping secondary symbionts: widespread horizontal transfer across and beyond the Aphidoidea. Mol. Ecol. **12**, 1061-1075. (10.1046/j.1365-294X.2003.01780.x) 12753224

[5] Henry LM, Maiden MCJ, Ferrari J, Godfray HCJ. 2015 Insect life history and the evolution of bacterial mutualism. Ecol. Lett. **18**, 516-525. (10.1111/ele.12425) 25868533

[6] Chiel E, Gottlieb Y, Zchori-Fein E, Mozes-Daube N, Katzir N, Inbar M, Ghanim M. 2007 Biotype-dependent secondary symbiont communities in sympatric populations of *Bemisia tabaci*. Bull. Entomol. Res. **97**, 407-413. (10.1017/S0007485307005159) 17645822

[7] Henry LM, Peccoud J, Simon JC, Hadfield JD, Maiden MJC, Ferrari J, Godfray HCJ. 2013 Horizontally transmitted symbionts and host colonization of ecological niches. Curr. Biol. **23**, 1713-1717. (10.1016/j.cub.2013.07.029) 23993843 PMC3980636

[8] Henry Y, Brechbühler E, Vorburger C. 2022 Gated communities: inter- and intraspecific diversity of endosymbionts across four sympatric aphid species. Front. Ecol. Evol. **10**, 1-7. (10.3389/fevo.2022.816184)

[9] Toju H, Fukatsu T. 2011 Diversity and infection prevalence of endosymbionts in natural populations of the chestnut weevil: relevance of local climate and host plants. Mol. Ecol. **20**, 853-868. (10.1111/j.1365-294X.2010.04980.x) 21199036

[10] Jaenike J, Unckless R, Cockburn SN, Boelio LM, Perlman SJ. 2010 Adaptation via symbiosis : recent spread of a Drosophila defensive symbiont. Science **329**, 212-215. (10.1126/science.1188235) 20616278

[11] Scarborough CL, Ferrari J, Godfray HCJ. 2005 Ecology: aphid protected from pathogen by endosymbiont. Science **310**, 1781. (10.1126/science.1120180) 16357252

[12] Hedges LM, Brownlie JC, O'Neill SL, Johnson KN. 2008 *Wolbachia* and virus protection in insects. Science **322**, 702. (10.1126/science.1162418) 18974344

[13] Oliver KM, Russell JA, Morant NA, Hunter MS. 2003 Facultative bacterial symbionts in aphids confer resistance to parasitic wasps. Proc. Natl Acad. Sci. USA **100**, 1803-1807. (10.1073/pnas.0335320100) 12563031 PMC149914

[14] Oliver KM, Smith AH, Russell JA. 2014 Defensive symbiosis in the real world - advancing ecological studies of heritable, protective bacteria in aphids and beyond. Funct. Ecol. **28**, 341-355. (10.1111/1365-2435.12133)

[15] Łukasik P, Guo H, van Asch M, Henry LM, Godfray HCJ, Ferrari J. 2015 Horizontal transfer of facultative endosymbionts is limited by host relatedness. Evolution (N Y) **69**, 2757-2766. (10.1111/evo.12767) 26332792

[16] Bright M, Bulgheresi S. 2010 A complex journey: transmission of microbial symbionts. Nat. Rev. Microbiol. **8**, 218-230. (10.1038/nrmicro2262) 20157340 PMC2967712

[17] Oliver KM, Moran NA, Hunter MS. 2005 Variation in resistance to parasitism in aphids is due to symbionts not host genotype. Proc. Natl Acad. Sci. USA **102**, 12 795-12 800. (10.1073/pnas.0506131102) PMC1200300 16120675

[18] Oliver KM, Degnan PH, Hunter MS, Moran NA. 2009 Bacteriophages encode factors required for protection in a symbiotic mutualism. Science **325**, 992-994. (10.1126/science.1174463) 19696350 PMC5473335

[19] Vorburger C, Ganesanandamoorthy P, Kwiatkowski M. 2013 Comparing constitutive and induced costs of symbiont-conferred resistance to parasitoids in aphids. Ecol. Evol. **3**, 706-713. (10.1002/ece3.491) 23533102 PMC3605857

[20] Niepoth N, Ellers J, Henry LM. 2018 Symbiont interactions with non-native hosts limit the formation of new symbioses. BMC Evol. Biol. **18**, 1-12. (10.1186/s12862-018-1143-z) 29530013 PMC5848548

[21] McLean AHC, Godfray HCJ. 2015 Evidence for specificity in symbiont-conferred protection against parasitoids. Proc. R. Soc. B **282**, 1-8. (10.1098/rspb.2015.0977) PMC4528558 26136451

[22] Smith AH et al. 2021 Does getting defensive get you anywhere?—Seasonal balancing selection, temperature, and parasitoids shape real-world, protective endosymbiont dynamics in the pea aphid. Mol. Ecol. **30**, 2449-2472. (10.1111/mec.15906) 33876478

[23] Gimmi E, Vorburger C. 2021 Strong genotype-by-genotype interactions between aphid-defensive symbionts and parasitoids persist across different biotic environments. J. Evol. Biol. **34**, 1944-1953. (10.1111/jeb.13953) 34695269 PMC9298302

[24] Weldon SR, Russell JA, Oliver KM. 2020 More is not always better: coinfections with defensive symbionts generate highly variable outcomes. Appl. Environ. Microbiol. **86**, 1-14. (10.1128/AEM.02537-19) PMC7028961 31862723

[25] Parker BJ, McLean AHC, Hrček J, Gerardo NM, Godfray HCJ. 2017 Establishment and maintenance of aphid endosymbionts after horizontal transfer is dependent on host genotype. Biol. Lett. **13**, 20170016 (10.1098/rsbl.2017.0016) 28566541 PMC5454236

[26] Sochard C, Morlière S, Toussaint G, Outreman Y, Sugio A, Simon JC. 2020 Examination of the success rate of secondary symbiont manipulation by microinjection methods in the pea aphid system. Entomol. Exp. Appl. **168**, 174-183. (10.1111/eea.12878)

[27] Lukasik P, Guo H, Van Asch M, Ferrari J, Godfray HCJ. 2013 Protection against a fungal pathogen conferred by the aphid facultative endosymbionts *Rickettsia* and *Spiroplasma* is expressed in multiple host genotypes and species and is not influenced by co-infection with another symbiont. J. Evol. Biol. **26**, 2654-2661. (10.1111/jeb.12260) 24118386

[28] McLean AHC, Godfray HCJ, Ellers J, Henry LM. 2019 Host relatedness influences the composition of aphid microbiomes. Environ. Microbiol. Rep. **11**, 808-816. (10.1111/1758-2229.12795) 31573138 PMC6900097

[29] Parker BJ, Hrček J, McLean AHC, Brisson JA, Godfray HCJ. 2021 Intraspecific variation in symbiont density in an insect–microbe symbiosis. Mol. Ecol. **30**, 1559-1569. (10.1111/mec.15821) 33512733

[30] Dykstra HR, Weldon SR, Martinez AJ, White JA, Hopper KR, Heimpel GE, Asplen MK, Oliver KM. 2014 Factors limiting the spread of the protective symbiont *Hamiltonella defensa* in *Aphis craccivora* aphids. Appl. Environ. Microbiol. **80**, 5818-5827. (10.1128/AEM.01775-14) 25015890 PMC4178609

[31] Sandström JP, Russell JA, White JP, Moran NA. 2001 Independent origins and horizontal transfer of bacterial symbionts of aphids. Mol. Ecol. **10**, 217-228. (10.1046/j.1365-294X.2001.01189.x) 11251800

[32] Russell JA, Moran NA. 2005 Horizontal transfer of bacterial symbionts: heritability and fitness effects in a novel aphid host. Appl. Environ. Microbiol. **71**, 7987-7994. (10.1128/AEM.71.12.7987-7994.2005) 16332777 PMC1317397

[33] Moran NA, Dunbar HE. 2006 Sexual acquisition of beneficial symbionts in aphids. Proc. Natl Acad. Sci. USA **103**, 12 803-12 806. (10.1073/pnas.0605772103) PMC1568928 16908834

[34] Rock DI, Smith AH, Joffe J, Albertus A, Wong N, O'Connor M, Oliver KM, Russell JA. 2018 Context-dependent vertical transmission shapes strong endosymbiont community structure in the pea aphid, *Acyrthosiphon pisum*. Mol. Ecol. **27**, 2039-2056. (10.1111/mec.14449) 29215202

[35] Cayetano L, Rothacher L, Simon JC, Vorburger C. 2015 Cheaper is not always worse: strongly protective isolates of a defensive symbiont are less costly to the aphid host. Proc. R. Soc. B **282**, 20142333 (10.1098/rspb.2014.2333) PMC4286048 25473015

[36] Kaech H, Dennis AB, Vorburger C. 2021 Triple RNA-Seq characterizes aphid gene expression in response to infection with unequally virulent strains of the endosymbiont *Hamiltonella defensa*. BMC Genomics **22**, 1-21. (10.1186/s12864-021-07742-8) 34134631 PMC8207614

[37] Oliver KM, Campos J, Moran NA, Hunter MS. 2008 Population dynamics of defensive symbionts in aphids. Proc. R. Soc. B **275**, 293-299. (10.1098/rspb.2007.1192) PMC2593717 18029301

[38] Leclair M, Polin S, Jousseaume T, Simon JC, Sugio A, Morlière S, Fukatsu T, Tsuchida T, Outreman Y. 2017 Consequences of coinfection with protective symbionts on the host phenotype and symbiont titres in the pea aphid system. Insect Sci. **24**, 798-808. (10.1111/1744-7917.12380) 27514019

[39] Clarke HV, Cullen D, Hubbard SF, Karley AJ. 2017 Susceptibility of *Macrosiphum euphorbiae* to the parasitoid *Aphidius ervi*: larval development depends on host aphid genotype. Entomologia Experimentalis et Applicata, 148-158

[40] Hall AAG, Steinbauer MJ, Taylor GS, Johnson SN, Cook JM, Riegler M. 2017 Unravelling mummies: cryptic diversity, host specificity, trophic and coevolutionary interactions in psyllid - parasitoid food webs. BMC Evol. Biol. **17**, 1-15. (10.1186/s12862-017-0959-2) 28587639 PMC5461677

[41] Degnan PH, Yu Y, Sisneros N, Wing RA, Moran NA. 2009 *Hamiltonella defensa*, genome evolution of protective bacterial endosymbiont from pathogenic ancestors. Proc. Natl Acad. Sci. USA **106**, 9063-9068. (10.1073/pnas.0900194106) 19451630 PMC2690004

[42] Lenhart PA, White JA. 2017 A defensive endosymbiont fails to protect aphids against the parasitoid community present in the field. Ecol. Entomol. **42**, 680-684. (10.1111/een.12419)

[43] Albittar L, Ismail M, Bragard C, Hance T. 2016 Host plants and aphid hosts influence the selection behaviour of three aphid parasitoids (Hymenoptera: Braconidae: Aphidiinae). Eur. J. Entomol. **113**, 516-522. (10.14411/eje.2016.068)

[44] Sanchez JA, La-Spina M, Michelena JM, Lacasa A, Hermoso de Mendoza A. 2011 Ecology of the aphid pests of protected pepper crops and their parasitoids. Biocontrol Sci. Technol. **21**, 171-188. (10.1080/09583157.2010.530641)

[45] Brodeurl J, Mcneil JN 1994 Seasonal Ecology of *Aphidius nigripes* (Hymenoptera: Aphidiidae), a Parasitoid of *Macrosiphum euphorbiae* (Homoptera: Aphididae). Environ. Entomol. **23**, 292-298. (10.1093/ee/23.2.292)

[46] Costopoulos K, Kovacs JL, Kamins A, Gerardo NM. 2014 Aphid facultative symbionts reduce survival of the predatory lady beetle *Hippodamia convergens*. BMC Ecol. **14**. (10.1186/1472-6785-14-5) PMC3936903 24555501

[47] Li Q, Fan J, Sun J, Zhang Y, Hou M, Chen J. 2019 Anti-plant defense response strategies mediated by the secondary symbiont *Hamiltonella defensa* in the wheat aphid *Sitobion miscanthi*. Front. Microbiol. **10**, 1-12. (10.3389/fmicb.2019.02419) 31708894 PMC6823553

[48] Foottit RG, Maw HEL, von Dohlen CD, Hebert PDN. 2008 Species identification of aphids (Insecta: Hemiptera: Aphididae) through DNA barcodes. Mol. Ecol. Res. **8**, 1189-1201. (10.1111/j.1755-0998.2008.02297.x) 21586006

[49] Heie OE. 1980 The Aphidoidea (Hemiptera) of Fennoscandia and Denmark. I. General Part. The families Mindaridae, Hormaphididae, Thelaxidae, Anoeciidae, and Pemphigidae. Fauna Entomol. Scand. 9:1–236.

[50] Degnan PH, Moran NA. 2008 Evolutionary genetics of a defensive facultative symbiont of insects: exchange of toxin-encoding bacteriophage. Mol. Ecol. **17**, 916-929. (10.1111/j.1365-294X.2007.03616.x) 18179430

[51] Hadfield JD, Nakagawa S. 2010 General quantitative genetic methods for comparative biology: phylogenies, taxonomies and multi-trait models for continuous and categorical characters. J. Evol. Biol. **23**, 494-508. (10.1111/j.1420-9101.2009.01915.x) 20070460

[52] R Core Team. 2021 R: A language and environment for statistical computing. R Foundation for Statistical Computing, Vienna, Austria. URL https://www.R-project.org/.

[53] McLean AHC, van Asch M, Ferrari J, Godfray HCJ. 2011 Effects of bacterial secondary symbionts on host plant use in pea aphids. Proc. R. Soc. B **278**, 760-766. (10.1098/rspb.2010.1654) PMC3030854 20843842

[54] Koga R, Tsuchida T, Fukatsu T. 2003 Changing partners in an obligate symbiosis: a facultative endosymbiont can compensate for loss of the essential endosymbiont Buchnera in an aphid. Proc. R. Soc. B **270**, 2543-2550. (10.1098/rspb.2003.2537) PMC1691542 14728775

[55] Fagan-Jeffries EP, Cooper SJB, Bertozzi T, Bradford TM, Austin AD. 2018 DNA barcoding of microgastrine parasitoid wasps (Hymenoptera: Braconidae) using high-throughput methods more than doubles the number of species known for Australia. Mol. Ecol. Res. **18**, 1132-1143. (10.1111/1755-0998.12904) 29791787

[56] Callahan BJ, McMurdie PJ, Rosen MJ, Han AW, Johnson AJA, Holmes SP. 2016 DADA2: high-resolution sample inference from Illumina amplicon data. Nat. Methods **13**, 581-583. (10.1038/nmeth.3869) 27214047 PMC4927377

[57] RStudio Team. 2020 RStudio: integrated development environment for R. RStudio, PBC, Boston, MA. URL http://www.rstudio.com/.

[58] Bates D, Mächler M, Bolker B, Walker S. 2015 Fitting linear mixed-effects models using lme4. J. Stat. Softw. **67**, 1-48. (10.18637/jss.v067.i01)

[59] Hothorn T, Bretz F, Westfall P. 2008 Simultaneous inference in general parametric models. Biom. J. **50**, 346-363. (10.1002/bimj.200810425) 18481363

[60] Wu T, Monnin D, Lee RAR, Henry LM. 2022 Local adaptation to hosts and parasitoids shape *Hamiltonella defensa* genotypes across aphid species. Zenodo. (10.5281/zenodo.7097415) PMC9597410 36285493

[61] Wu T, Monnin D, Lee RAR, Henry LM. 2022 Local adaptation to hosts and parasitoids shape *Hamiltonella defensa* genotypes across aphid species. Figshare. (10.6084/m9.figshare.c.6251410) PMC9597410 36285493

